# Does Particulate Matter Modify the Association between Temperature and Cardiorespiratory Diseases?

**DOI:** 10.1289/ehp.9266

**Published:** 2006-07-27

**Authors:** Cizao Ren, Gail M. Williams, Shilu Tong

**Affiliations:** 1 School of Public Health, Queensland University of Technology, Kelvin Grove, Brisbane, Queensland, Australia; 2 School of Population Health, University of Queensland, Herston, Brisbane, Queensland, Australia

**Keywords:** air pollution, interaction, mortality, particulate matter, temperature

## Abstract

**Background:**

A number of studies have shown that both temperature and air pollution are associated with health outcomes. In assessing air pollution effects, temperature is usually considered a confounder. However, only a few recent studies considered air pollution as confounders while assessing temperature effects. Few studies are available on whether or not air pollution modifies the temperature–disease relationship.

**Methods:**

In this study, we used three parallel Poisson generalized additive models to examine whether particulate matter < 10 μm in aerodynamic diameter (PM_10_) modified the effects of minimum temperature on cardiorespiratory morbidity and mortality in Brisbane, Australia.

**Results:**

Results show that PM_10_ statistically significantly modified the effects of temperature on respiratory and cardiovascular hospital admissions, all nonexternal-cause mortality, and cardiovascular mortality at different lags. The enhanced adverse temperature effects were found at higher levels of PM_10_, but no clear evidence emerged for interactive effects on respiratory and cardiovascular emergency visits. Three parallel models produced similar results, which strengthened the validity of findings.

**Conclusion:**

We conclude that it is important to evaluate the modification role of air pollution in the assessment of temperature-related health impacts.

The nature and magnitude of the association between temperature and human health has been increasingly recognized (Basu and Samet 2002; Martens 1997; Patz et al. 2000; Samet et al. 1998). Both hyperthermia and hypothermia are generally linked to cardiorespiratory morbidity or mortality (Braga et al. 2001; 2002; Kunst et al. 1993). The patterns of temperature–morbidity/mortality vary across regions, with J-, U-, or V-shapes most commonly observed (Basu and Samet 2002; Braga et al. 2002; Patz et al. 2000). In many regions of the world, death rates in winter are usually higher than those in summer, even though heat waves can cause excess deaths (Braga et al. 2002; McMichael et al. 2001). Seasonal variation in morbidity and mortality may also reflect factors beyond weather, including seasonal patterns of respiratory infections. Consequently, assessments of the effect of weather on human health have usually controlled for seasonality and sometimes for influenza epidemics (Schwartz et al. 2004).

Meanwhile, numerous studies have shown that air pollution is consistently associated with adverse health effect (Bell et al. 2004; Dominici et al. 2006; Samet et al. 2000). However, the role of air pollution is often ignored in assessing the health effects of temperature variability, except in a few recent studies adjusting for air pollution (O’Neill et al. 2003; Rainham and Smoyer-Tomic 2003). None of the previous studies have explored whether exposure to air pollution modifies the association between temperature and health outcomes. If substantial effect modification exists, an inappropriately specified model may result in bias. First, it may be inappropriate to consider air pollution only as a confounder in the assessment of the association between temperature and health outcomes, because air pollution may make people more vulnerable to the effects of temperature variability. Second, some studies have shown that temperature may modify the associations between air pollution and cardiorespiratory diseases (Choi et al. 1997; Katsouyanni et al. 1993; Ren and Tong 2006; Roberts 2004). There is often symmetry in modification—air pollution modifies temperature and then temperature modifies air pollution—but the magnitudes are likely to differ. Finally, the true magnitude of the association between temperature and health outcomes may be obscured if air pollution is an effect modifier of the relationship. In this study we used three parallel time-series models to explore whether particulate matter < 10 μm in aerodynamic diameter (PM_10_) modified the effects of temperature on cardiorespiratory hospital admissions, emergency visits, and mortality in Brisbane, Australia, during the period 1996–2001.

## Materials and Methods

### Data collection

The data sets consisted of concurrent daily time series of health outcomes, weather, and air pollution collected in Brisbane City from 1 January 1996, to 31 December 2001. Brisbane City is the capital of Queensland, Australia, with a subtropical climate. In 2001, there were 0.89 million residents in Brisbane City (Brisbane City Council 2006).

Health outcome data in this study were provided by the Queensland Department of Health and comprised cardiovascular hospital admissions (CHA), cardiovascular emergency visits (CEV), cardiovascular mortality (CM), respiratory hospital admissions (RHA), respiratory emergency visits (REV), and all non-external-cause mortality (NECM). This analysis excluded respiratory mortality due to limited daily counts (mean, 1.5; range, 0–8). Discharge diagnosis was classified according to *International Classification of Diseases, 9th Revision* (ICD-9; World Health Organization 1975) (used until July 1999) or *10th Revision* (ICD-10; World Health Organization 1992) codes: respiratory diseases (ICD-9: 460–519 or ICD-10: J00–J99), cardiovascular diseases (ICD-9: 390–448 or ICD-10: I00–I79), and external causes (ICD-9: E800–E999 or ICD-10: S00–U99). Influenza (ICD-9: 487.0–487.8 or ICD-10: J10–J11) was excluded from respiratory diseases, but occurrence of influenza outbreak was considered as a potential confounder in the data analysis. All cases were local residents of Brisbane City. The notification of incidence and mortality is a statuary requirement under the Health Act 1937 for all public and private hospitals, nursing homes and pathology services in Queensland. The Queensland Department of Health is responsible for data collection, management, and analysis (Queensland Government 2001).

Daily meteorologic data were supplied by the Australian Bureau of Meteorology (http://www.bom.gov.au/), including daily minimum temperature, relative humidity and rainfall for the period of this study. Air pollution data included ambient 24-hr average concentrations of PM_10_ and ozone. All air pollution data were regularly recorded at a central monitoring site and provided by the Queensland Environmental Protection Agency (http://www.epa.qld.gov.au/).

### Data analysis

Poisson generalized additive models (GAM) were employed to explore the associations of temperature and PM_10_ with health outcomes. This assumed that the daily number of counts had an overdispersed Poisson distribution [*E* (*Y_t_* *=* μ*_t_*), var(*Y_t_*) = φμ*_t_*] (Dominici et al. 2004). GAM allows nonparametric smoothing functions to account for potentially nonlinear effects of confounding factors on the dependent variable, such as seasonal variation and weather conditions (Hastie and Tibshirani 1990). We used days of calendar time with a cubic smoothing function to control for the confounding effect of seasonality. We controlled for short-term fluctuation using day of the week as a factor. Other potential confounders, such as relative humidity and influenza outbreaks, were also adjusted for.

Before exploring effect modification of PM_10_ on the temperature-health relationship, we used an independent model to explore the patterns of the relationship between temperature and health outcomes. The independent model is described below (Daniels et al. 2000; Hastie and Tibshirani 1990; Insightful Corporation 2001):


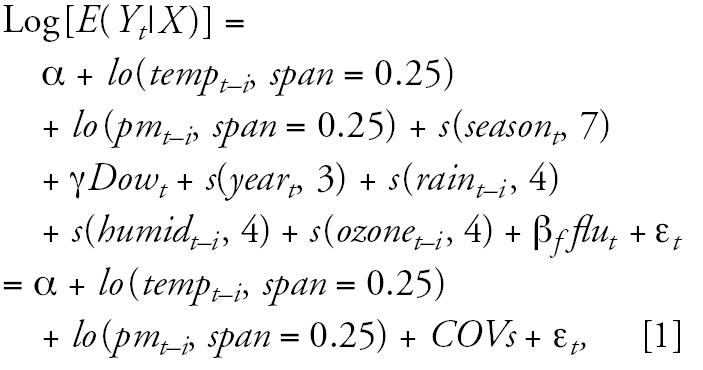


where *t* refers to the day of the observation; *i* refers to lags; *E*(*Y_t_*|*X* ) denotes estimated daily case counts on day *t; s*(·) and *lo*(·) separately denote the cubic smoothing spline and LOESS smooth functions, respectively; α is the intercept term; *temp_t_*_−_*_i_* is 24-hr minimum temperature on day *t*−*i; pm_t_*_−_*_i_* is PM_10_ on day *t*−*i; season_t_* denotes seasonality using days of calendar time. In accordance with the literature (Daniels et al. 2000), we used 7 degrees of freedom (df) per year for season so that little information from time scales longer than 2 months was included. *Dow_t_* is the day of week on day *t,* and γ is a vector of coefficients. The variables *rain_t_*_−_*_i_, humid_t_*_−_*_i_,* and *ozone_t_*_−_*_i_* refer to rainfall, relative humidity at 0900 hr and ozone on day *t*−*i*, respectively; *flu_t_* represents the occurrence of influenza epidemics. Because > 99% of days only have 0 or 1 influenza cases, influenza was categorized as a dummy variable (0 cases, ≥ 1 cases on day *t*). β*_f_* is the coefficient for influenza; ɛ*_t_* is the residual. *COVs* represents all other covariates in the model.

Then we used three GAM models to assess whether PM_10_ modified the association of temperature with health outcomes: a nonparametric bivariate response model, a nonstratification model, and a stratification model (Ren and Tong, in press; Roberts 2004). We used a bivariate model to explore visually the combining effects of both temperature and PM_10_ with health outcomes. This was undertaken using a nonparametric smoothing function without linear assumptions that the two predictors linearly depend on outcomes. We used a nonstratification model quantitatively to examine the association of both above predictors with health outcomes with a linear assumption by including an interaction term of temperature and PM_10_ as continuous functions. We used a stratification model quantitatively to assess the associations of temperature with health outcomes across PM_10_ levels by including an interaction term of temperature and PM_10_ in which PM_10_ was categorized into two levels. The three models are described in detail below.

First, we used the nonparametric bivariate response model to identify the joint effects of minimum temperature and PM_10_ on health outcomes. This can capture the relationship between independent and dependent variables without the need for strong assumptions (Hastie and Tibshirani 1990). This model provided a picture of the joint pattern of two predictors (temperature and PM_10_) on the dependent variable (each of cardiorespiratory morbidities and mortalities). Therefore, it can be used to observe whether or not there is an interactive effect of two continuous predictors on the dependent variable (Greenland 1993; Hastie and Tibshirani 1990). We modified Equation 1 to include a bivariate term for temperature and PM_10_ as follows (Insightful Corporation 2001; Ren and Tong, in press; Roberts 2004):


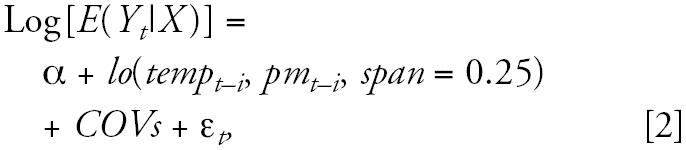


where *lo*(*temp_t_*_−_*_i_*, *pm_t_*_−_*_i_*) means joint effect of temperature and PM_10_ and *COVs* was the same as model 1.

Second, we used a nonstratification model, assuming a linear relationship, to estimate the interactive effects of PM_10_ and minimum temperature on health outcomes. We added an interaction term to estimate increment in cardiorespiratory mortality/morbidity per unit change in ambient PM_10_ and minimum temperature, as follows:


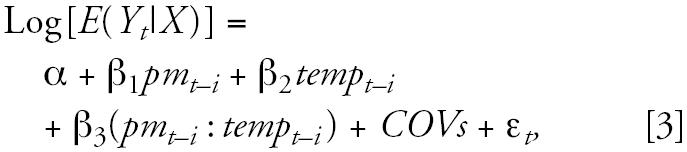


where β_1_ denotes the increment in mortality/morbidity per unit increase in ambient PM_10_ level, β_2_ denotes the increment in mortality/morbidity per unit increase in temperature level, and β_3_ estimates the interactive effect of PM_10_ and temperature on health outcomes after adjustment for all other covariates. *COVs* was the same as in model 1.

Finally, we applied a stratification model to examine whether the effects of temperature on health outcomes were heterogeneous across different levels of PM_10_. We categorized PM_10_ into two levels (low and high) and then examined whether temperature effects varied across levels of PM_10_. To assess effect modification in the high end of the temperature range, we used separate data sets to fit this model: one data set with the whole range of temperatures and another database with temperatures ≥ 19.3 °C (75th percentile). We slightly modified Equation 3 as follows:


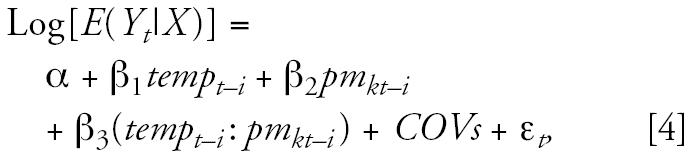


where *pm_kt_* represents levels of PM_10_, *temp_t_*_−_*_i_* : *pm_kt_*_−_*_i_* represents the interaction term of temperature and levels of PM_10_, and other covariates were the same as in Equation 3. Because PM_10_ was categorized into just two levels, each of *pm_kt_* and *temp_t_*_−_*_i_* : *pm_kt_*_−_*_i_* has one coefficient denoted by β_2_ and β_3_, respectively. *COVs* was the same as in model 1.

S-plus software (version 6.2) was used in the data analyses (Chambers and Hastie 1993; Insightful Corporation 2001). To reduce potential bias caused by convergence, we used stricter criteria: 1.0 × 10^−10^ for both the local score algorithm and the backfitting algorithm (Dominici et al. 2002). We used the S-plus function *gam.exact* (Dominici et al. 2004; Internet-based Health and Air Pollution Surveillance System 2006) to correct the potential underestimation of the coefficient’s standard error due to concurvity (Ramsay et al. 2003). Furthermore, analyses were restricted to days that contained values for all covariates in each model (> 87% of observations).

## Results

**Figure 1:**
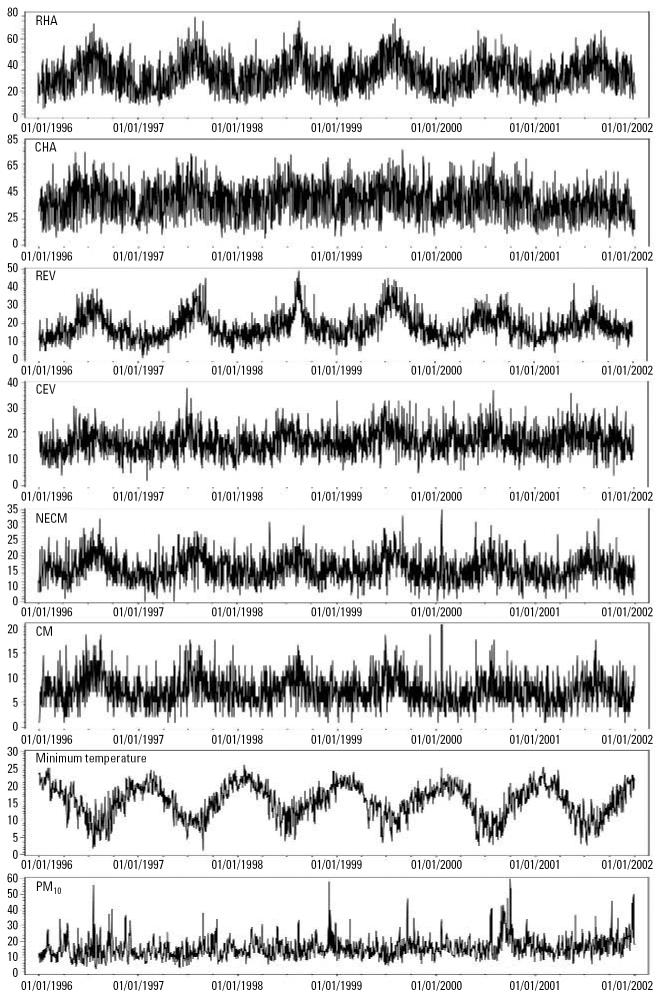
Time-series distributions of PM_10_, minimum temperature, and health outcomes from 1996 to 2001 in Brisbane. The panels represent the distributions of RHA, CHA, REV, CEV, NECM, CM (number of daily cases), minimum temperature (°C), and PM_10_ (μg/m), from top to bottom.

We examined the distributions of each of the dependent variables, temperature, and PM_10_ by time. The results show that there were strong seasonal patterns for RHA, CHA, REV, CEV, NECM, CM, and temperature, but the PM_10_ pattern was less obvious (Figure 1). There were also apparent short-term fluctuations in health outcomes, minimum temperature, and the concentration of PM_10_.

Table 1 provides summary statistics for individual health outcomes and explanatory variables. The results show considerable variation in each variable, ranging from 6 to 77 for RHA, 7 to 90 for CHA, 1 to 48 for REV, 2 to 38 for CEV, 5 to 42 for NECM, 1 to 31 for CM, 1.2 to 26 °C for temperature, and 2.5 to 60.0 μg/m^3^ for PM_10_.

**Figure 2:**
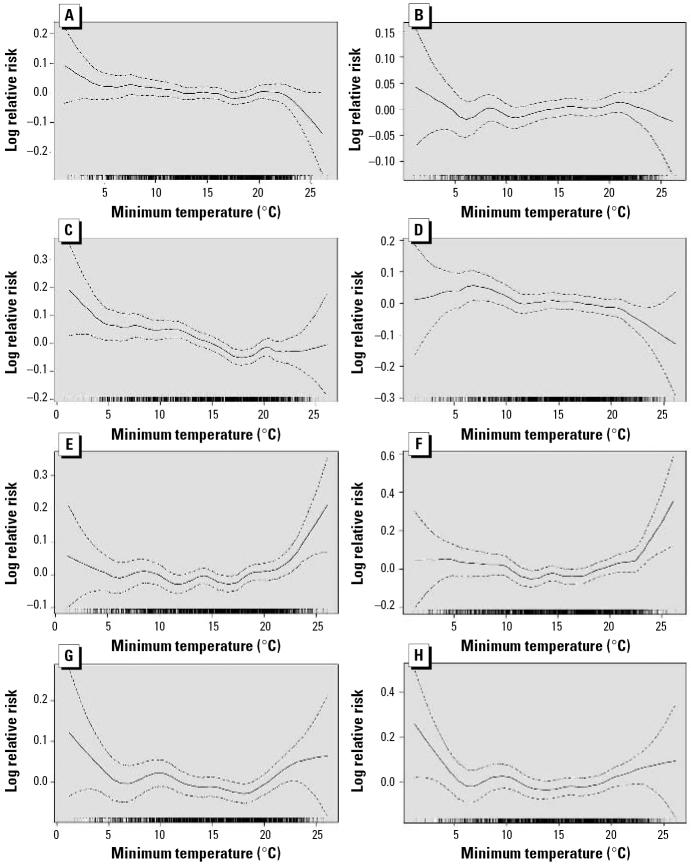
Temperature–morbidity/mortality relationship. (*A*) RHA, (*B*) CHA, (*C*) REV, (*D*) CEV, (*E*) NECM, and (*F*) CM, all on current day, and (*G*) NECM and (*H*) CM, both at lag 2. Solid lines represent point estimates and dashed lines for 95% confidence intervals.

In the first model, there were inverse relationships between temperature and various measures of cardiorespiratory morbidity except for CHA, which showed a slight positive relationship (Figure 2). The patterns were similar at lags of 0, 1, or 2 days (RHA, CHA, REV, and CEV). Patterns of temperature effect on current day for morbidity and current day and lag 2 for mortality are presented (Figure 2). However, the relationships between temperature and cardiorespiratory mortality (NECM and CM) differed from the morbidity outcomes and varied by lengths of lag. For the current day, the associations of temperature with NECM and CM were relatively slight when the temperature was between 0 and 20ºC and then increased quickly, but at lags of 1 and 2 days, the associations first decreased and then leveled off or slightly increased. Hence, the relationship between temperature and mortality forms a J- or U-shaped pattern, depending on the lag time (Basu and Samet 2002; Braga et al. 2002; Patz et al. 2000).

**Figure 3:**
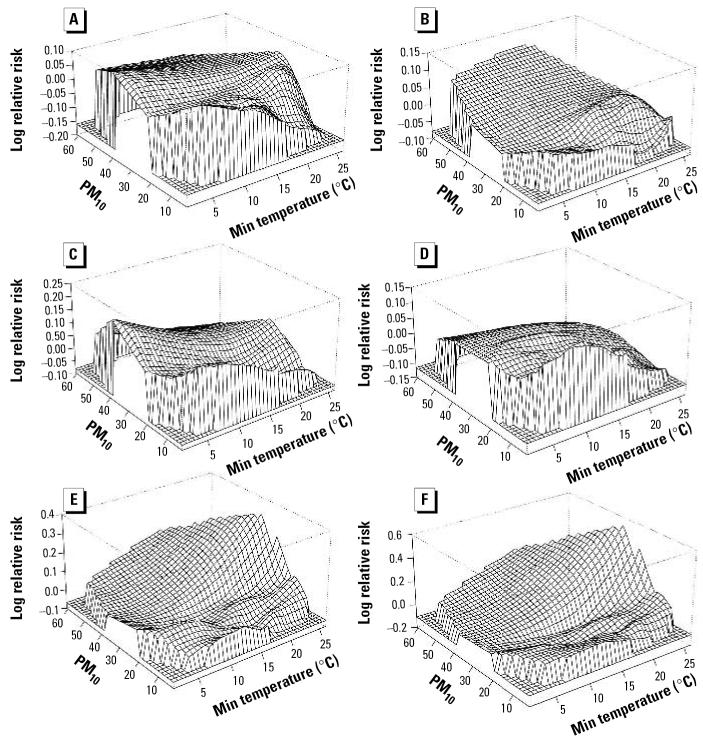
Bivariate response surfaces of minimum (Min) temperature and PM_10_ on health outcomes on current day for (*A*) RHA, (*B*) CHA, (*C*) REV, (*D*) CEV, (*E*) NECM, and (*F*) CM.

To explore potential effect modification of PM_10_ and temperature on cardiorespiratory morbidity/mortality, we separately fitted bivariate response surface models (model 2) with individual health outcomes at each of three lags (0, 1, and 2 days). The results show interactive effects of PM_10_ and temperature on RHA, REV, NECM, and CM at all time points, less so for CHA and CEV. Figure 3 illustrates the joint effects of PM_10_ and temperature on each health outcome (RHA, CHA, REV, CEV, NECM, and CM) for the current day. Temperature effects were modified by levels of PM_10_ for RHA, REV, NEMC, and CM, less so for CHA and CEV. Favorable temperature effects disappeared when PM_10_ was above the mean or median (15.84 or 14.8 μg/m^3^) for RHA and REV, but adverse temperature effects appeared for NECM and CM when PM_10_ was above the mean or median. CHA was similar to RHA. The bivariate response surfaces differed from the independent model results, showing that the association between temperature and mortality changed with PM_10_. In fact, what at first appeared to be a J-shaped relationship in the independent model (model 1) became an approximately linear relationship when the joint effect of PM_10_ and temperature was taken into account (model 2). There were inverse linear associations between minimum temperature and morbidity or mortality at low levels of PM_10_ (< 20 μg/m^3^). However, at higher levels of PM_10_, the association between temperature and mortality was positively linear, whereas the associations with the various morbidity measures were weak.

Because no obvious J- or U-shaped patterns of the temperature-health relationship were observed in bivariate response models, we separately fitted nonstratification models (model 3) using each of the cardiorespiratory morbidity/mortality measures as a response variable with the same set of predictors at each of the lags (Table 2). The results indicate statistically significant interactive effects between temperature and PM_10_ on RHA, CHA, NECM, and CM at different lags. For example, PM_10_ modified the effects of temperature on RHA and CM at all lags, but modified the effects of temperature on NECM at lags of 0 and 2 days and CHA at lag 2, marginally at lag 0. No significant interactions were found for REV and CEV. The results were similar to those from model 2. Because the estimated effects of temperature variability differed with PM_10_ levels, we present the estimated coefficients of model 3 instead of relative risks (Table 2).

To test sensitivity of changes in degrees of freedom related to the number of categories used for covariates, we refitted model 3 using 12 df for *season_t_* (each month), 6 df for year (each year), and 8 df for rain, relative humidity, and ozone instead of the original df. Results show that increases in df changed the modeling outcomes only minimally.

Both the bivariate response surface and nonstratification models suggest that the effects of temperature on cardiorespiratory morbidity/mortality varied with levels of PM_10_. We then fitted the stratification model (model 4) to examine heterogeneity of temperature associations with health outcomes across different strata of PM_10_, defined as greater than or less than the mean level (15.8 μg/m^3^) of PM_10_. There were statistically significant interactions for RHA, CHA, NECM, and CM at different lags, but not for CEV and REV. Table 3 shows the percent changes in cardiorespiratory morbidity/mortality per 10°C increase in minimum temperature across the different levels of PM_10_. Temperature effects on cardiorespiratory morbidity/mortality varied across the different levels of PM_10_. For most lags and most health outcomes, the percent changes were higher when PM_10_ levels were higher. For morbidity measures, this meant that the inverse association with temperature was less extreme for high PM_10_; for mortality measures, the association with minimum temperature became positive at high PM_10_ levels. For example, when minimum temperature increased by 10°C (using data with the full range of temperature), there was a decrease in RHA of 7.2% and 1.0% on the current day, with low and high PM_10_ levels, respectively. To examine the association at the high end of the temperature range with health outcomes, we also fitted model 4 using data sets constrained to the highest quartile (≥ 19.3°C) of temperature with the same cutoffs for temperature as the whole database. The pattern was even stronger when analyzed in the high-temperature data set (Table 3).

## Discussion

In this study, we used three parallel time-series approaches to examine whether PM_10_ modified the association between temperature and cardiorespiratory morbidity/mortality. Results show that PM_10_ modified the effects of temperature on respiratory hospital admissions, cardiovascular hospital admissions, all non-external-cause mortality, and cardiovascular mortality in different lags. In particular, more adverse outcomes were evident with increasing temperature when PM_10_ levels also increased. However, there were no significant interactive effects between temperature and PM_10_ on respiratory and cardiovascular emergency visits. Three parallel models produced similar results.

In this study we used different health outcomes to examine consistency of findings. However, the findings from different health outcomes for the same observed groups varied somewhat. Reasons for this might include different age distributions, different events, and clinical features. For example, for respiratory hospital admissions and emergency visits, acute upper respiratory infection, pneumonia, and asthma were the dominant causes, and a high proportion of cases were identified in children. For cardiovascular hospital admission and emergency visits, angina pectoris, artrial fibrillation and flutter, and chronic ischemic heart diseases were the main causes, and elderly persons comprised most of theses cases. For MECN and CM, acute myocardial infarction, chronic ischemic heart diseases, and stroke were the main causes, and again elderly persons comprised most cases. Several studies have shown that age and preexisting diseases modify the air pollution–health association (Dubowsky et al. 2006; O’Neill et al. 2003). In considering the variation in findings for different health effects, we note that this study is designed to detect short-term effects of air pollution (within a few days). Mechanisms related to high temperature or PM_10_ that precipitate acute illness may be different or have different magnitudes, depending on the underlying diseases.

Temperature and air pollution are generally highly correlated in many places (Holgate et al. 1999), and they may interact symmetrically to affect health outcomes. Although whether air pollution modifies temperature estimates has not been investigated so far, several studies have found evidence that temperature may modify the relationship between air pollution and morbidity or mortality (Choi et al. 1997; Katsouyanni et al. 1993; Ren and Tong, in press; Roberts 2004). For example, Katsouyanni et al. (1993) examined whether air pollution and ambient temperature had synergistic effects on excess mortality during the 1987 “heat wave” in Athens. They found a statistically significant modification of temperature on the association between exposure to sulphur dioxide and total excess mortality, although the main effect of this pollutant was not statistically significant. Roberts (2004) investigated the interaction between daily particulate air pollution and daily mean temperature on mortality in Cook County, Illinois, and Allegheny County, Pennsylvania, using data for 1987–1994. The study found that temperature modified the association between PM_10_ and mortality, but the results were sensitive to the number of degrees of freedom. Our recent study also found that temperature significantly modified the association between PM_10_ and health outcomes (Ren and Tong, in press). These findings support the hypothesis that PM_10_ might modify the relationship between temperature and health outcomes.

It is biologically plausible that PM_10_ modifies the effects of temperature on cardiorespiratory diseases. A range of studies have shown that PM_10_ is consistently associated with health outcomes (Dominici et al. 2006; Samet et al. 2000). Exposure to PM_10_ may directly affect airways through inhalation, including upper airways, bronchiole, and alveolus. The exposure could modulate the automatic nervous system and might further influence the cardiovascular system (Gordon 2003; Jeffery 1999). Some studies have shown that PM_10_ is associated with decreased heart rate variation (Creason et al. 2001; Gold et al. 2000). Marked temperature changes also affect physiological and psychological stresses (Gordon 2003), which could aggravate preexisting diseases. Therefore, both high ambient temperature and high ambient PM_10_ may interact to synergistically effect human morbidity/mortality.

Each of the three models used in this study has inherent advantages and disadvantages. The bivariate response surface model is a flexible approach to show the patterns of two continuous predictors on the dependent variable and explore whether potential interaction exists without a rigid assumption of linearing between predictors and the dependent variable (Greenland 1993; Hastie and Tibshirani 1990). However, this model can not provide parametric estimates for exposure effects; therefore, it may be difficult to judge whether interactive effects exist and also to compare the results from different studies. The nonstratification parametric model includes a pointwise product of two continuous variables. Parametric estimates for both predictors and their pointwise product can be obtained (Chambers and Hastie 1993). However, the linear assumption between the dependent variable and both continuous predictors is not necessarily met in all situations, especially for temperature and air pollution in a multisite study with variation in study populations. Moreover, the estimated coefficients of both predictors can not be simply interpreted as the main effects (Table 2) (Chambers and Hastie 1993). The stratification parametric model provides parametric estimates, which can be easily interpreted as main effects and interaction. The parametric estimates can be used in a meta-analysis. However, because the effect of one continuous predictor generally changed with another predictor level (Figure 3), the selection of cutoffs is still a challenge, especially in comparing the results from different studies.

We explored a marker of air pollution as a modifier of the relationship between temperature and cardiovascular morbidity/mortality. We used an independent model (model 1) and a bivariate response model (model 2) to examine the patterns of temperature with several health outcomes (RHA, CHA, REV, CEV, NECM, and CM). A J-shaped pattern was observed only for NECM and CM on the current day but not for other measures of cardiorespiratory morbidity. No obvious J-shaped pattern was observed in the bivariate response surface models, suggesting that the interaction between PM_10_ and temperature may play an important role in model fit. Therefore, when modeling the health effects of air pollution and/or temperature, an interaction between these two factors should be carefully considered. Many studies have shown J-, U-, or V-shaped patterns of the temperature–health relationship (Basu and Samet 2002; Braga et al. 2002; Patz et al. 2000). The different patterns observed may be caused by different climate conditions across studies. For example, Braga et al. (2002) reported that greater variability of summer and winter temperature was associated with larger effects for hot and cold days, respectively, on respiratory deaths. However, Brisbane has a subtropical climate, with few extremely cold days (for example, the mean minimum temperature was 15.4°C and the lowest temperature was 1.2°C during the study period).

This study is an ecologic design, and misclassifications are possible for both health outcomes and exposure. Because a broad classification of diseases (cardiovascular, respiratory, and nonexternal classification of diseases) was used, we do not believe that misclassification for health outcomes is likely to be substantial. We used air pollution from one central monitoring site to represent individual exposure to PM_10_ and this might result in misclassification. However, previous studies have shown that central fixed-site measurements may be treated as surrogates for personal exposure (Kim et al. 2005) and bias from the monitoring data might not be severe. Although some families in Brisbane have access to air conditioning, thus reducing exposure to high temperature when indoors, this effect is believed minimal due to the small proportion of houses with air conditioning as well as the outdoor lifestyle of Queensland residents.

There are two major strengths of this study. First, this is, to our knowledge, the first study to examine whether PM_10_ modifies the association of temperature and a range of cardiorespiratory morbidity/mortality measures. Second, we performed three parallel statistical models with multiple health outcomes, and they produced similar findings, which strengthens the validity of findings.

However, this study also has two key limitations. First, caution is needed in interpreting any time-series study within a single location. This study was carried out in a single city with a subtropical climate, and 6 years of data are not extensive. Therefore, the results of this study may be difficult to generalize to other places. Second, this study is an ecologic design, in which bias from exposure measurement errors might occur to some degree due to lack of individual information.

Overall, we found statistically significant interactive effects of PM_10_ and temperature on respiratory and cardiovascular hospital admission, all nonexternal-cause mortality, and cardiovascular mortality at different lags in Brisbane during the study period. The temperature effects were more adverse at high levels of PM_10_. These findings may have important implications in the assessment of health effects of temperature and the development of strategies and policies for controlling and preventing temperature-related deaths and diseases. However, it is necessary to determine whether a consistent finding could be found in other settings.

## Figures and Tables

**Table 1:** Summary statistics for health outcomes, air pollutants, and meteorologic conditions.

Variable	Mean	Minimum	25th Percentile	Median	75th Percentile	Maximum
RHA (*n*)	33.16	6	23	33	42	77
CHA (*n*)	39.32	7	30	39	48	90
REV (*n*)	18.39	1	13	17	23	48
CEV (*n*)	17.17	2	14	17	20	38
NECM (*n*)	15.86	5	13	15	18	42
RM (*n*)	1.45	0	0	1	2	8
CM (*n*)	7.26	1	5	6	9	31
PM_10_ (μg/m^3^)	15.84	2.5	11.9	14.8	18.7	60
Temperature (°C)	15.42	1.2	12.0	16.0	19.3	26
Humidity (%)	65.21	12.0	57	65	74	100
Ozone (ppb)	11.26	0	7	10	15	45
Rainfall (mm)	3.038	0	0	0.0	0.8	163

**Table 2:** Coefficients for main and interactive effects of minimum temperature and PM_10_ on different health outcomes using model 3.

Lag^a^	Variable	RHA	CHA	REV	CEV	NECM	CM
Lag0	Temperature	−0.012019*	−0.003942	−0.009377*	−0.003304	−0.008374**	−0.008193
	PM_10_	−0.004296**	0.000150	−0.000887	0.000737	−0.004022	−0.004565
	Interaction	0.000471*	0.000232#	0.000135	−0.000015	0.000534*	0.000603**
Lag1	Temperature	−0.009196*	−0.0003767	−0.010039**	−0.003636	−0.005321	−0.010081
	PM_10_	−0.002474**	0.000028	−0.004209	−0.001248	−0.002182	−0.003927
	Interaction	0.000339**	0.000124	0.000271	0.000116	0.000322#	0.000556**
Lag2	Temperature	−0.008950*	−0.009764*	−0.009406*	−0.008885**	−0.008556#	−0.015128**
	PM_10_	−0.004229**	−0.002946	−0.003440	−0.003383	−0.005177	−0.007141
	Interaction	0.000413*	0.000259**	0.000280#	0.000210	0.000441**	0.000809**

a Lag refers to 0, 1, or 2 days.

* *p* < 0.01.

** *p* < 0.05.

# *p* < 0.10.

**Table 3:** Percent change (%) in cardiorespiratory morbidity/mortality per 10°C increase in temperature across the levels of PM_10_.

Lag^a^	Variable	RHA (95% CI)	CHA (95% CI)	REV (95% CI)	CEV (95% CI)	NECM (95%CI)	CM (95% CI)
Whole range of temp							
Lag0	PM Low^b^	−7.2 (−11.3 to −2.9)	−2.3 (−6.3 to 1.7)	−6.8 (−12.1 to −1.1)	−2.2 (−7.8 to 3.5)	−1.4 (−7.3 to 4.8)	−0.9 (−9.8 to 7.9)
	PM High	−1.0 (−5.0 to 3.2)	1.1 (−2.5 to 4.7)	−6.6 (−11.5 to −1.4)	−4.5 (−9.7 to 0.6)	2.8 (−2.7 to 8.7)	4.6 (−3.4 to 12.6)
Lag1	PM Low	−2.9 (−7.3 to 1.6)	−2.6 (−6.7 to 1.4)	−3.6 (−9.2 to 2.3)	−2.1 (−7.8 to 3.7)	−0.2 (−6.2 to 6.0)	−1.4 (−10.2 to 7.5)
	PM High	−2.4 (−6.5 to 1.8)	−1.0 (−4.6 to 2.5)	−5.3 (−10.3 to −0.1)	−1.2 (−6.3 to 3.9)	0.6 (−4.8 to 6.4)	0.0 (−7.9 to 8.0)
Lag2	PM Low	−3.2 (−7.6 to 1.3)	−8.2 (−12.2 to −4.2)	−3.9 (−9.5 to 2.0)	−6.7 (−12.4 to −1.0)	−3.9 (−9.6 to 2.2)	−3.6 (−12.5 to 5.2)
	PM High	−0.4 (−4.5 to 3.8)	−3.5 (−7.1 to 0.1)	−4.4 (−9.4 to 1.9)	−4.2 (−9.3 to 1.0)	1.1 (−4.3 to 6.9)	0.9 (−7.0 to 8.8)
Temp ≥ 19.3°C							
Lag0	PM Low	−29.2 (−40.6 to −15.5)	−4.7 (−19.2 to 10.0)	−7.4 (−27.2 to 17.8)	−13.2 (−33.8 to 7.8)	9.9 (−12.9 to 38.5)	0.8 (−31.3 to 34.1)
	PM High	5.2 (−17.0 to 33.4)	−3.8 (−22.8 to 15.6)	4.5 (−23.1 to 42.0)	−94.2 (−118.6 to 69.1)	14.0 (−15.4 to 53.7)	44.1 (0.8 to 89.2)
Lag1	PM Low	−7.6 (−23.4 to 11.5)	0.7 (−13.7 to 15.3)	1.2 (−20.2 to 28.3)	−14.9 (−35.3 to 6.0)	35.1 (7.0 to 70.5)	18.9 (−13.6 to 52.4)
	PM High	−8.1 (−27.6 to 16.6)	12.2 (−7.4 to 32.1)	51.5 (11.1 to 106.6)	1.5 (−25.7 to 29.6)	14.8 (−15.4 to 55.8)	26.2 (−17.3 to 71.6)
Lag2	PM Low	−12.8 (−27.4 to 4.8)	15.9 (1.1 to 30.8)	−24.8 (−40.8 to −4.6)	8.0 (−13.0 to 29.4)	13.2 (−10.2 to 42.6)	6.7 (−25.4 to 39.8)
	PM High	−19.2 (−36.4 to 2.6)	12.8 (−6.7 to 32.7)	−8.8 (−33.3 to 24.7)	29.4 (−51.8 to 117.5)	37.6 (1.9 to 85.8)	23.2 (−19.1 to 67.4)

Abbreviations: CI, confidence interval; Temp, minimum temperature.

a Lag refers to 0, 1, or 2 days.

b PM indicates PM_10_, categorized into low and high levels using mean of PM_10_ as a cutoff.
